# Targeting Mitochondrial Metabolism in Prostate Cancer with Triterpenoids

**DOI:** 10.3390/ijms22052466

**Published:** 2021-02-28

**Authors:** Kenza Mamouni, Georgios Kallifatidis, Bal L. Lokeshwar

**Affiliations:** 1 Georgia Cancer Center, Augusta University, Augusta, GA 30912, USA; GKALLIFATIDIS@AUGUSTA.EDU; 2 Research Service, Charlie Norwood VA Medical Center, Augusta, GA 30904, USA; 3 Department of Biological Sciences, Augusta University, Augusta, GA 30912, USA

**Keywords:** mitochondrial metabolism, Warburg effect, prostate cancer, triterpenoids

## Abstract

Metabolic reprogramming is a hallmark of malignancy. It implements profound metabolic changes to sustain cancer cell survival and proliferation. Although the Warburg effect is a common feature of metabolic reprogramming, recent studies have revealed that tumor cells also depend on mitochondrial metabolism. Due to the essential role of mitochondria in metabolism and cell survival, targeting mitochondria in cancer cells is an attractive therapeutic strategy. However, the metabolic flexibility of cancer cells may enable the upregulation of compensatory pathways, such as glycolysis, to support cancer cell survival when mitochondrial metabolism is inhibited. Thus, compounds capable of targeting both mitochondrial metabolism and glycolysis may help overcome such resistance mechanisms. Normal prostate epithelial cells have a distinct metabolism as they use glucose to sustain physiological citrate secretion. During the transformation process, prostate cancer cells consume citrate to mainly power oxidative phosphorylation and fuel lipogenesis. A growing number of studies have assessed the impact of triterpenoids on prostate cancer metabolism, underlining their ability to hit different metabolic targets. In this review, we critically assess the metabolic transformations occurring in prostate cancer cells. We will then address the opportunities and challenges in using triterpenoids as modulators of prostate cancer cell metabolism.

## 1. Introduction

At diagnosis, ~90% of prostate cancers (PCa) are organ-confined or locally advanced [1,2]. Based on clinical stage and prostate-specific antigen (PSA) levels, the therapeutics for localized PCa are active surveillance, local radiotherapy, or prostatectomy. With the spread of the disease outside the prostate, with or without treatment for localized tumors, androgen deprivation therapy (ADT) by surgical or chemical castration is the recommended therapeutic strategy. Unfortunately, the response is usually transient, and most patients will develop resistance to ADT and progress towards castration-resistant prostate cancer (CRPC) in ~18–36 months [3]. Indeed, ADT can suppress hormone-naïve prostate cancer, but prostate cancer adapts to survive under castration levels of androgen [4]. The implicated mechanisms include androgen receptor (AR) point mutations, AR overexpression, changes of androgen biosynthesis, constitutively active ligand-independent AR splice variants, and changes of androgen cofactors [5]. Studies of AR in CRPC revealed that AR can still be involved in CRPC, and it remains a potential target to treat CRPC. Enzalutamide is a second-generation AR antagonist effective in patients with CRPC which, unlike first-generation antagonists, interrupts several key components of AR signaling: androgen binding to the AR, nuclear translocation of activated AR, and binding of activated AR with DNA [6,7]. However, CRPC is still incurable and can develop drug resistance. Treatment failure is often associated with the development of even more aggressive subtypes, such as neuroendocrine prostate cancer (NEPC) [8,9].

Under aerobic conditions, normal cells process glucose, first to pyruvate via glycolysis in the cytosol and then to carbon dioxide in the mitochondria. Under anaerobic conditions, glycolysis is favored, and relatively little pyruvate is dispatched to the oxygen-consuming mitochondria [10,11]. Otto Warburg first observed an anomalous characteristic of cancer cell energy metabolism even in oxygenated cells. Cancer cells can reprogram their glucose metabolism, and thus their energy production, by limiting their energy metabolism primarily to glycolysis, leading to a state that has been termed “aerobic glycolysis” [10,11]. While not maximizing ATP production, aerobic glycolysis allows cancer cells to efficiently convert glucose into biomass (e.g., nucleotides, amino acids, and lipids) for cell growth and proliferation [12,13]. As opposed to Warburg’s theory, most—if not all—cancer cells rely on functional mitochondria for their survival [14].

The metabolic phenotype of prostate epithelial cells is unique, with changes occurring during tumor onset and progression from prostatic intraepithelial neoplasia (PIN) to metastasis. Normal prostate epithelial cells are highly dependent on glycolysis and produce citrate from glucose. The tricarboxylic acid (TCA) cycle does not function efficiently to produce large amounts of citrate. During transformation, PCa cells progressively reactivate mitochondrial oxidative phosphorylation (OXPHOS) with increased glucose metabolism and decreased citrate production. In PCa, AR reprograms global cellular metabolic pathways, including both aerobic glycolysis and mitochondrial respiration, along with de novo lipogenesis, thereby supporting the metabolic and biosynthetic demands of PCa cells [15,16].

## 2. Mitochondrial Metabolism

### 2.1. The Tricarboxylic Acid (TCA) Cycle

The TCA cycle is a series of enzyme-catalyzed chemical reactions that form a key part of aerobic respiration in all living cells under aerobic conditions. During glycolysis, glucose is oxidized to pyruvate in the cytosol. Under aerobic conditions, pyruvate is transported into the mitochondrial matrix, where it is converted into acetyl-CoA, which fuels the TCA cycle [17,18]. Metabolites generated from the catabolism of nutrients, such as lipids, carbohydrates, and proteins, are catabolized through different pathways that converge in the synthesis of acetyl-CoA, a crucial fuel for the TCA cycle, to generate energy in the form of ATP [18]. In fatty acid β-oxidation, long-chain fatty acids are catabolized in the mitochondrial matrix to ultimately produce three energy storage molecules per round of oxidation, one NADH, one FAD(H2), and one acetyl-CoA molecule [19,20]. Furthermore, glutamine is catabolized via glutaminolysis to form glutamate, which can be converted in the mitochondria to α-ketoglutarate, a constituent of the TCA cycle [21].

### 2.2. Oxidative Phosphorylation

Mitochondria, as the powerhouse of the cell, are the main source of adenosine triphosphate (ATP) generated via oxidative phosphorylation (OXPHOS) [18,22]. The OXPHOS metabolic pathway generates ATP by the transport of electrons to a series of transmembrane protein complexes in the mitochondrial inner membrane, known as the electron transport chain (ETC) [23]. NADH, FADH2, and succinate act as electron donors [23]. As the electrons pass through the multiprotein ETC complexes I to IV, protons are pumped from the mitochondrial matrix into the intermembrane space by complexes I, III, and IV [23]. When OXPHOS is active, protons flow from the inner intermembrane space back into the mitochondrial matrix through complex V, ATP synthase, driving the synthesis of ATP [23]. Oxygen acts as the terminal electron acceptor [23].

### 2.3. AMP-Activated Pprotein Kinase (AMPK): The Guardian of Metabolism

Cells continuously adapt their metabolism to meet their energy needs based on the availability of nutrients and on their capacity to produce ATP [24]. Upon changes in energy availability and changes in the ATP-to-ADP or ATP-to-AMP ratio, AMP-activated protein kinase (AMPK) is activated by an allosteric mechanism that stimulates its kinase activity. Indeed, elevated adenosine monophosphate (AMP) and adenosine diphosphate (ADP) levels act as molecular red flags, signaling to the cell that energy needs are not met. In response, AMPK stimulates catabolic pathways needed to bolster ATP reserves while slowing cellular anabolic processes to reduce energy consumption [25]. Specifically, AMPK regulates the phosphorylation of key proteins in multiple pathways, including mTOR complex 1 (mTORC1) [26], lipid homeostasis [27], glycolysis [28], and mitochondrial homeostasis [29]. Excellent recent reviews have focused on the regulation of various aspects of metabolism by AMPK [24,30].

Autophagy is a process by which cellular components such as macromolecules and organelles, as well as pathogens, are recycled by a specialized cellular machinery. The targets, which are subject to autophagic degradation, are engulfed in membranous structures called autophagosomes. The latter fuse with lysosomes forming autophagolysosomes where the cargo becomes degraded [24]. Autophagy mainly serves two distinct functions: the turnover of old or damaged molecules and the replenishment of nutrient stores during times of starvation. Autophagy is promoted by AMPK, the key energy sensor that regulates cellular metabolism to maintain energy homeostasis [31]. Conversely, autophagy is inhibited by the mammalian target of rapamycin (mTOR), a central cell-growth regulator that integrates growth factor and nutrient signals [32]. Under glucose starvation, AMPK promotes autophagy by directly activating Ulk1 (Unc-51 like autophagy activating kinase 1) through phosphorylation of Serine 317 and Serine 777. Under nutrient sufficiency, high mTOR activity prevents Ulk1 activation by phosphorylating Ulk1 Serine 757 and disrupting the interaction between Ulk1 and AMPK [32]. This coordinated phosphorylation is important for Ulk1 in autophagy induction [32]. Therefore, AMPK and mTOR are described as the Yin and the Yang of cellular nutrient sensing and growth control [33].

## 3. Metabolic Characteristics of Normal Prostatic Epithelium and Prostate Cancer

### 3.1. Normal Prostate Epithelial Cell Metabolism

**Figure 1:**
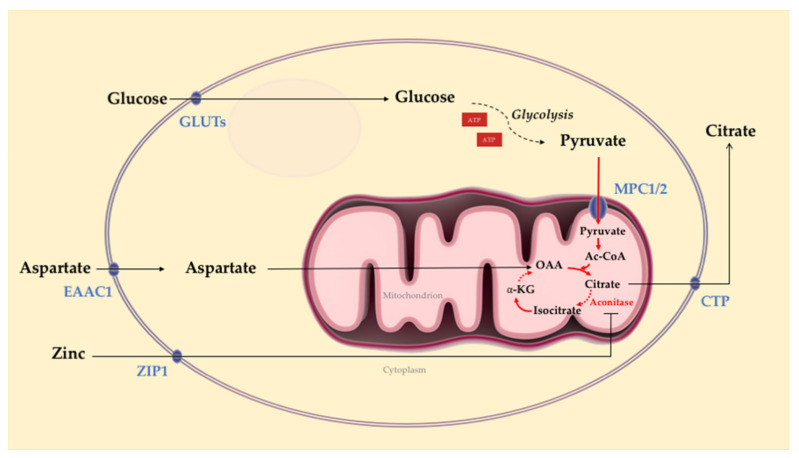
Overview of normal prostate epithelial cell metabolism. Regulated by androgen receptor (AR) signaling, normal prostate epithelium assimilates glucose and aspartate to generate and secrete citrate and produce most ATP via glycolysis. Intracellular accumulation of zinc through active transport results in the inhibition of aconitase, preventing the conversion of citrate into isocitrate. As a result, citrate accumulates and is secreted. Solid arrows represent single metabolic steps, and dashed arrows represent simplified multistep processes. Ac-CoA: acetyl-coenzyme A; α-KG: α-ketoglutarate; OAA: oxaloacetate; GLUTs, glucose transporters; MPC1/2: mitochondrial pyruvate carrier 1 and 2; EAAC1: excitatory amino-acid carrier 1; ZIP1: zinc transporter 1; CTP: citrate transporter. Solid arrows: single metabolic step; dashed arrows: simplified multistep processes (Figure adapted from Bader et al., 2020 [35] and Twum-Ampofo et al., 2016 [43]).

Regulated by AR signaling, the metabolism in normal prostatic epithelial cells is particularly interesting as it demonstrates a unique metabolic feature compared to most other tissues in the body [34,35,36]. The prostate’s unique metabolic processes are well adapted to fulfill the major function as a secretory tissue to generate prostatic fluid comprised of a high concentration of citrate along with zinc, lipids, and kallikrein enzymes, including prostate-specific antigen (PSA) [37]. Prostate metabolism is highly specialized to assimilate glucose and aspartate to generate and secrete citrate in prostatic fluid (Figure 1). Indeed, prostatic tissue secretes high levels of citrate [38] and zinc [39]. Zinc is an essential regulatory cofactor for >300 enzymes and transcription factors [38,40]. Citrate is hypothesized to act as a chelator of calcium and zinc and a scavenger of free radicals [41,42]. In the normal prostate epithelium, citrate excretion is maintained by inhibition of aconitase, a tricarboxylic acid (TCA) cycle enzyme responsible for converting citrate to isocitrate [39,43]. The mitochondrial citrate is then transported to the cytoplasm and ultimately excreted into seminal fluid [39,43]. The aconitase inhibition is mediated by high zinc levels [39], regulated by an abundance of ZIP1 zinc transporters in prostatic epithelial cells [44].

### 3.2. Prostate Tumor Metabolism

**Figure 2:**
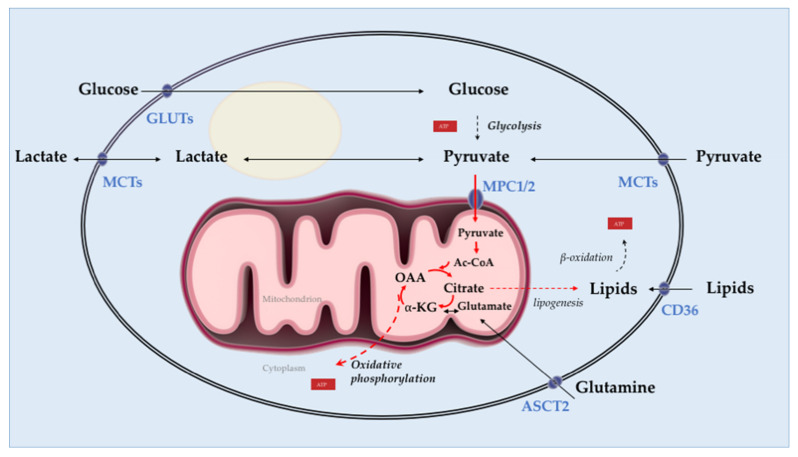
Overview of prostate adenocarcinoma cell metabolism. In prostate tumors, androgen receptor mediates metabolic reprogramming characterized by reduced glycolysis, enhanced mitochondrial oxidative phosphorylation, and enhanced lipogenesis. In contrast to the normal prostatic epithelium, citrate is oxidized in the tricarboxylic acid (TCA) cycle in prostate cancer (PCa) cells and is used to fuel lipogenesis. Lipids promote energy (ATP) synthesis through β-oxidation. Further, pyruvate is the primary fuel in the TCA cycle of PCa cells. Activation of AR signaling enhances glutamine uptake and metabolic assimilation via upregulation of the glutamine transporter ASCT2. Solid arrows represent single metabolic steps, and dashed arrows represent simplified multistep processes. Ac-CoA: acetyl-coenzyme A; α-KG: ⍺-ketoglutarate; OAA: oxaloacetate; GLUTs: glucose transporters; MPC1/2: mitochondrial pyruvate carrier 1 and 2; MCTs: monocarboxylate transporters; ASCT2: alanine-serine-cysteine transporter 2; CD36: cluster of differentiation 36, fatty acid transporter. Solid arrows: single metabolic step; dashed arrows: simplified multistep processes.

Intriguingly, PCa loses the ability to concentrate zinc and citrate, which results in the transformation of prostate epithelial cells from benign and citrate-secreting to malignant and citrate-oxidizing cells, a hallmark of PCa [43]. Indeed, zinc is depleted, and as a result, citrate does not accumulate and gets oxidized in the TCA cycle to fuel lipogenesis [45]. In addition to the changes in zinc and citrate metabolism, AR-mediated metabolic reprogramming in PCa results in reduced glycolysis, enhanced mitochondrial oxidative phosphorylation, and increased lipogenic capacity compared with normal prostate tissue [46]. In contrast to most other solid tumors, PCa is less glycolytic and relies predominantly on oxidative phosphorylation compared to adjacent normal tissue [45]. Although AR signaling does not seem to have a direct role in the loss of zinc transporters [35,47], it fuels proliferation through regulation of central metabolic pathways including, but not limited to, glycolysis [15,48], oxidative phosphorylation [49,50], glutamine uptake [51,52] and lipogenesis [53,54] (Figure 2).

## 4. Targeting Mitochondrial Metabolism of Prostate Cancer with Triterpenoids

Triterpenoids are metabolites of isopentenyl pyrophosphate (IPP) oligomers and comprise the largest natural plant products group, with over 20,000 known members [55] Triterpenoids are found in various plants, including seaweeds and wax-like coatings of various fruits and medicinal herbs, including apples, cranberries, figs, olives, mistletoe, lavender, oregano, rosemary, and thyme [56,57,58]. Triterpenoids are also isolated from invertebrates [59,60], and fungi [61]. Due to their multiple biological activities, triterpenoids are extensively evaluated for their potential use in human pathophysiology [62,63]. Triterpenoids are synthesized from IPP via the 30-carbon intermediate squalene and include sterols, steroids, and triterpenoid saponins [64]. Native triterpenoids, in particular, oleanane and ursane representatives, are of interest for researchers due to their availability and multiple biological activities, including antibacterial [65], anti-inflammatory [66], and antitumor effects [56,67,68]. Triterpenoids regulate various transcription and growth factors, inflammatory cytokines, and intracellular signaling pathways involved in cancer cell proliferation, apoptosis and tumor angiogenesis in a variety of cancer cell types, including prostate cancer [69,70] Interestingly, an increasing number of studies [71,72,73,74] have reported the impact of triterpenoids on tumor cell metabolism. Those studies shed light on their mechanisms of action and their potential as anti-cancer metabolic modulators. Here, we review triterpenoids with the ability to modulate metabolism in prostate cancer.

### 4.1. Ursolic Acid (UA)

Lodi et al. [75] studied the metabolic pathways of PCa cell lines treated with ursolic acid with an untargeted metabolomics approach incorporating magnetic resonance spectroscopy (MRS) and mass spectrometry (MS). Interestingly, combinations of some phytochemicals with UA (UA + curcumin and UA + resveratrol) decreased ATP levels and inhibited the growth/survival of both mouse (HMVP2, established from tumors of Hi-Myc mice [76]) and human (PC-3 and LNCaP) PCa cell lines [75]. Interestingly, both of these combinations produced a synergistic inhibition of HMVP2 cells in vivo in an allograft tumor model when administered in the diet [75]. Metabolic analysis of isotope-labeled glutamine revealed a decreased glutamine uptake in cells treated with UA in combination with curcumin or resveratrol [75]. UA, either alone or in combination with curcumin, decreased the protein level of the glutamine transporter ASCT2 [75]. Mechanistically, the combination treatments (UA+ curcumin and UA+ resveratrol) increased AMPK and decreased mTORC1 activity [75].

### 4.2. Oleanolic Acid (OA)

The main characteristic of the Warburg effect is characterized by a metabolic switch from mitochondrial oxidative phosphorylation to aerobic glycolysis [77]. Liu et al. [78] analyzed the effect of OA on aerobic glycolysis by measuring glucose uptake, lactate production, and oxygen consumption. The authors found that OA treatment reduced glucose consumption and lactate production and enhanced oxygen consumption in PC-3 cells, indicating that OA suppresses aerobic glycolysis. Furthermore, the authors analyzed the pyruvate kinase expression (PK) isoforms PKM1 and PKM2. PK catalyzes phosphoenolpyruvate (PEP) conversion to pyruvate, which is the last step of the glycolytic pathway. In contrast to the constitutively active PKM1 isoform, PKM2 relies on the binding of fructose-1,6-bisphosphate (FBP) for the transition to an active stage [79]. PKM2 is expressed mainly in actively proliferating cells during embryogenesis and in cancer. The lower enzymatic activity of PKM2 than PKM1 allows cancer cells to rewire their metabolism and utilize the intermediates of glycolysis towards biosynthetic pathways, which is essential for proliferating cells. On the contrary, the PKM1 isoform is associated with decreased tumor cell proliferation [79]. Interestingly, Liu et al. showed that OA decreased PKM2 expression levels and increased PKM1 expression levels in PC-3 cells in a dose and time-dependent manner. The *PKM* gene encodes two subtypes of pyruvate kinase (PK) [80], PKM1 and PKM2, key rate-limiting enzymes for glycolysis that catalyze the phosphorylation of phosphoenolpyruvate (PEP) and adenosine diphosphate (ADP) to produce pyruvate and adenosine triphosphate (ATP) [80]. Increasing PKM2 activity or switching mRNA splicing from PKM2 to PKM1 can suppress the Warburg effect, and consequently, compromise tumor growth [79,81]. Moreover, OA decreased mTOR activation, as assessed by reduced levels of p-mTOR^Ser2448^. The mTOR increases PKM2 protein levels by stimulating c-Myc-dependent hnRNPA1 and hnRNPA2 expression [82], both of which are responsible for the exclusive spicing of PKM mRNA [83]. The data showed that OA reduced the expression level of c-Myc, and its targets hnRNAP1 and hnRNAP2 in PC3 cells [78]. To conclude, the authors identified for the first time that OA could suppress aerobic glycolysis by suppressing PKM2 expression and affected PKM mRNA splicing through mTOR/c-Myc/hnRNP signaling [78]. Another study [84] described that OA induced AMPK activation in PC-3 cells in a dose and time-dependent manner, as assessed by the increase in p-AMPK^Thr172^ levels. Interestingly, OA treatment decreased lipid synthesis in PC3 cells. Furthermore, OA treatment decreased fatty acid synthase (FASN) protein levels [84], a critical enzyme in the lipogenic pathway that is frequently found to be overexpressed in cancer cells [85]. Radioactive assays revealed that OA decreased de novo lipid synthesis, shown as a reduced incorporation rate of [3H] acetyl-CoA [84].

### 4.3. Nummularic Acid (NA)

The triterpenoid nummularic acid (NA) was isolated for the first time in 2007 from the plant *Evolvulus nummularius* [86] and is extensively used for the treatment of jaundice, pneumonia, and malaria in countries like Morocco, India and, China [87]. NA treatment significantly reduced the proliferation and migration of PCa cell lines DU145 and C4-2 in a time- and dose-dependent manner and induced apoptosis, as revealed by the induction of PARP and Caspase 3 cleavage [88]. Mechanistically, NA treatment increased the ADP/ATP ratio to activate AMPK (phosphorylation of AMPK α subunit at Thr172 residue). NA simultaneously increased acetyl CoA carboxylase phosphorylation and decreased pS6 phosphorylation, two major substrates of AMPK [88]. The authors assessed the impact of NA treatment on lactate production in real-time and cellular oxidative phosphorylation (OXPHOS) by measuring the extracellular acidification rate (ECAR) and the oxygen consumption rate (OCR), respectively. The data showed that NA decreased OCR and increased ECAR, indicating reduced OXPHOS and a metabolic shift in favor of glycolysis. Non-targeted metabolomics analysis by ALEX-CIS-GC-TOF-MS indicated that NA increased glucose levels, glucose-6-phosphate, and 3-phosphoglycerate in DU145 and C4-2 PCa cell lines, suggesting an induction in glycolytic rate. The authors also screened TCA metabolites and found that NA treatment increased α-ketoglutarate (α-KG) and glutamate levels, suggesting that the cells utilized alternative fuels to compensate for decreased oxidative phosphorylation. Therefore, the authors concluded that NA induced an energy crisis by modulating glycolysis, TCA, and glutamine metabolism, via AMPK activation.

### 4.4. Plectranthoic Acid

**Figure 3:**
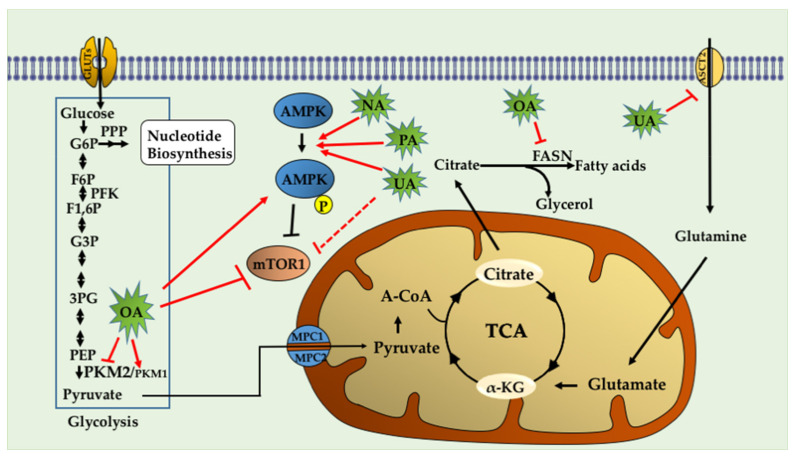
Triterpenoids target metabolic pathways in prostate cancer. Triterpenoids activate the cellular energy sensor AMPK, promoting catabolic pathways and ATP synthesis, but inhibit the anabolic pathways required for cell growth and proliferation. OA inhibits the enzyme fatty acid synthase (FASN), which catalyzes long-chain fatty acid synthesis. OA also induces expression of PKM1 and reduces expression of PKM2, interfering with the biosynthetic pathways that are sustained by the expression of PKM2 in cancer cells. PCa cells utilize glutamine, which is transformed to glutamate and then to α-ketoglutarate. The latter is shuttled into the TCA cycle. Ursolic acid (UA) inhibits the glutamine transporter ASCT2 and glutamine uptake, and thus the energetic pathways mediated by glutamine. OA: Oleanolic Acid, UA: Ursolic acid, NA: Nummularic acid, PA: Plectranthoic acid, G6P: Glucose-6-phosphate, F6P: Fructose-6-phosphate, F1,6P: Fructose 1,6-bisphosphate, PFK: phosphofructokinase, G3P: Glyceraldehyde-3-phosphate, 3-PG: 3-phosphoglycerate, PEP: phosphoenolpyruvate, PPP: pentose phosphate pathway.

Akhtar et al. isolated for the first time the triterpenoid plectranthoic acid (PA) from *Ficus microcarpa*, a traditional plant with antidiabetic properties [89]. PA exhibited significant anti-proliferative activity against PCa cell lines such as PC-3, DU145, and CW22R*V*1 [89]. PA treatment suppressed mTOR/S6K signaling and induced apoptosis in PCa cells in an AMPK-dependent manner [89]. In silico modeling, employed to determine potential interactions between PA and AMPK, indicated the γ subunit of AMPK as the preferred binding site for PA. Interestingly, the binding sites of PA were compared to the AMP binding sites and, it was found that PA does not compete with AMP for AMPK binding. AMPK requires phosphorylation of the kinase at the Thr^172^ residue by upstream regulators such as LKB1 and CaMKKβ [90]. PA stimulated AMPK activity independently of LKB1, observed in both LKB1 null DU145 and LKB1 positive PC3 cells. Comparative studies of PA with the widely used AMPK activator metformin suggest different mechanisms of actions of these compounds. Metformin is also reported to bind to γ-subunit of AMPK; however, unlike AMP, metformin is not a direct allosteric AMPK activator [91]. Although the binding of PA to γ subunit of AMPK results in allosteric activation of the kinase, further studies are warranted to determine the mechanism through which PA interacts with AMPK and induces the phosphorylation and activation of AMPK. In conclusion, the authors identified PA as a potent AMPK activator. (Figure 3, Table 1 and Table 2)

## 5. Conclusions and Future Directions

Our current understanding of the complexity of PCa metabolism and the molecular pathways involved is rapidly evolving. The metabolic characterization and treatment of PCa could be an important approach to provide personalized treatment for the disease. From the studies presented here, it is apparent that triterpenoids are unique in their potential to target metabolic pathways. There may exist a better preventive or therapeutic potential in the synergistic action of these triterpenoids. The ability of triterpenoids to target multiple metabolism pathways might be advantageous in limiting compensatory signaling feedback loops and cross-talk between cellular pathways. This is a strategy to be further explored in future studies. However, in most studies, individual compounds have been tested in in vitro prostate cancer models. A significant limitation of these studies is that cell culture media compositions do not reflect the complex and heterogenic metabolic milieu of tumors [100]. Tumors, including prostate cancer, are characterized by spatial heterogeneity of metabolic preferences, which is attributed to the metabolic crosstalk between cancer cells and the tumor microenvironment [100,101,102]. Moreover, spatial nutrient gradients and the resulting differences in their availability, and the oxygen limitation in hypoxic areas, can shape the metabolic phenotypes and contribute to the metabolic heterogeneity observed in tumors [100,101,102]. Future studies must address the effects of triterpenoids in vivo. Another important consideration is that triterpenoids are insoluble in aqueous media, limiting their bioavailability, a critical requirement for in vivo efficacy. One approach to enhance the water solubility of triterpenoids could be the structural modification of naturally occurring compounds to generate more polar analogs. Several other possibilities for improving the hydrophilicity of triterpenoids include the design and generation of formulations containing cyclodextrin complexes, liposomes, colloids, micelles and nanoparticles [103,104,105]. Additionally, both pharmacokinetic and pharmacodynamic studies are equally important in evaluating and improving the bioavailability and biological effects of these triterpenoids for clinical application.

## Figures and Tables

**Table 1:** Characteristics of prostate cancer (PCa) cell lines discussed in the review.

Cell Line	Origin	AR Status/Androgens Sensitivity	References
**Mouse cell line**			
HMVP2	Established from Hi-Myc mice	AR+, androgen-responsive	[76]
**Human cell lines**			
RWPE-1	Benign cell line from peripheral prostate zone	AR+, androgen-responsive	[92]
PC-3	Bone metastasis	AR-, androgen irresponsive	[93]
DU-145	Brain metastasis	AR-, androgen irresponsive	[94]
LNCaP	Lymph node metastasis	AR+, androgen-responsive	[95]
C4-2	Derived from castrated mice bearing LNCaP tumors	AR+, both androgen-independent and androgen- sensitive	[96,97]
CW22RV1	Derived from androgen-dependent CWR22 xenograft	AR+, androgen-independent. Express both wild-type and AR-v7 variant of AR.	[98]

**Table 2:** Effects of triterpenoids on metabolic pathways of prostate cancer (PCa) cell lines.

Triterpenoid (Name and Chemical Structure)	Cell Line	Metabolic Effects	References
Ursolic acid 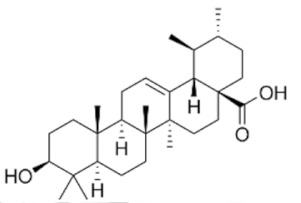	HMPV2, PC-3 and LNCaP	↓ mTORC1 ↑AMPK ↓ATP ↓ASCT2 (glutamine transporter)	[99]
Oleanolic acid 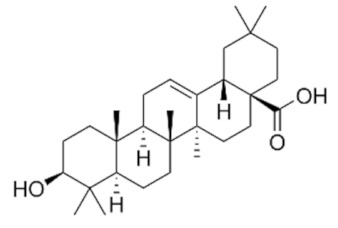	PC-3	↓ glucose uptake ↓lactate levels ↑O_2_ consumption rate ↑ PKM1 ↓ PKM2 ↓p-mTOR (Ser2448) ↑p-AMPK(Thr172) ↓lipogenesis (↓FASN)	[78,84]
Nummularic acid 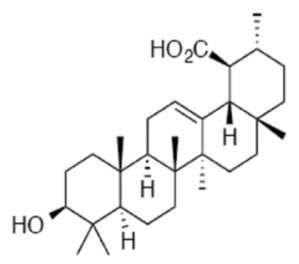	DU145, C4-2	↓ ATP/ADP ratio ↑ AMPK ↑Acetyl CoA ↓PS6 phosphorylation ↑ glycolysis ↓ O_2_ consumption rate ↑glucose-6 phosphate ↑α- ketoglutarate ↑glutamate	[88]
Plectranthoic acid 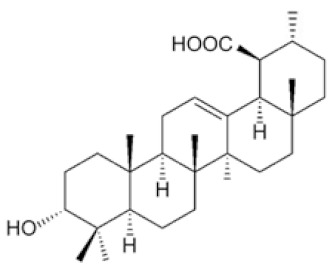	DU145, PC3	↓ mTOR/S6K ↑ p-AMPK(Thr172)	[89]

## Data Availability

Not applicable.

## Abbreviations

PCa	prostate cancer
PSA	prostate-specific antigen
ADT	androgen deprivation therapy
CRPC	castration-resistant prostate cancer
NEPC	neuroendocrine prostate cancer
ROS	reactive oxygen species
PIN	prostatic intraepithelial neoplasia
TCA	tricarboxylic acid
OXPHOS	oxidative phosphorylation
AR	androgen receptor
IPP	isopentenyl pyrophosphate
ECAR	extracellular acidification rate
OCAR	oxygen consumption rate
ALEX-CIS-GC-TOF-MS	automated liner exchange-cold injection system-gas chromatography-time of flight mass spectrometer-mass spectrometry

## References

[1] Yap T.A., Smith A.D., Ferraldeschi R., Al-Lazikani B., Workman P., De Bono J.S. (2016). Drug discovery in advanced prostate cancer: Translating biology into therapy. Nat. Rev. Drug Discov..

[2] Siegel R.L., Miller K.D., Jemal A. (2020). Cancer statistics, 2020. CA Cancer J. Clin..

[3] Watson P.A., Arora V.K., Sawyers C.L. (2015). Emerging mechanisms of resistance to androgen receptor inhibitors in prostate cancer. Nat. Rev. Cancer.

[4] Mostaghel E.A., Montgomery B., Nelson P.S. (2009). Castration-resistant prostate cancer: Targeting androgen metabolic pathways in recurrent disease. Urol. Oncol. Semin. Orig. Investig..

[5] Fujita K., Nonomura N. (2019). Role of Androgen Receptor in Prostate Cancer: A Review. World J. Men’s Health.

[6] Tran C., Ouk S., Clegg N.J., Chen Y., Watson P.A., Arora V., Wongvipat J., Smith-Jones P.M., Yoo D., Kwon A. (2009). Development of a Second-Generation Antiandrogen for Treatment of Advanced Prostate Cancer. Science.

[7] Scott L.J. (2018). Enzalutamide: A Review in Castration-Resistant Prostate Cancer. Drugs.

[8] Beltran H., Rickman D.S., Park K., Chae S.S., Sboner A., Macdonald T.Y., Wang Y., Sheikh K.L., Terry S., Tagawa S.T. (2011). Molecular Characterization of Neuroendocrine Prostate Cancer and Identification of New Drug Targets. Cancer Discov..

[9] Chang A.J., Autio K.A., Roach M., Scher H.I. (2014). High-risk prostate cancer-classification and therapy. Nat. Rev. Clin. Oncol..

[10] Warburg O. (1956). On the Origin of Cancer Cells. Science.

[11] Warburg O. (1956). On respiratory impairment in cancer cells. Science.

[12] Heiden M.G.V., Cantley L.C., Thompson C.B. (2009). Understanding the Warburg Effect: The Metabolic Requirements of Cell Proliferation. Science.

[13] Heiden M.G.V., Locasale J.W., Swanson K.D., Sharfi H., Heffron G.J., Amador-Noguez D., Christofk H.R., Wagner G., Rabinowitz J.D., Asara J.M. (2010). Evidence for an Alternative Glycolytic Pathway in Rapidly Proliferating Cells. Science.

[14] Heiden M.G.V., DeBerardinis R.J. (2017). Understanding the Intersections between Metabolism and Cancer Biology. Cell.

[15] Massie C.E., Lynch A., Ramos-Montoya A., Boren J., Stark R., Fazli L., Warren A., Scott H., Madhu B., Sharma N. (2011). The androgen receptor fuels prostate cancer by regulating central metabolism and biosynthesis. EMBO J..

[16] Audet-Walsh É., Dufour C.R., Yee T., Zouanat F.Z., Yan M., Kalloghlian G., Vernier M., Caron M., Bourque G., Scarlata E. (2017). Nuclear mTOR acts as a transcriptional integrator of the androgen signaling pathway in prostate cancer. Genes Dev..

[17] Melkonian E.A., Schury M.P. (2020). Biochemistry, Anaerobic Glycolysis.

[18] Vakifahmetoglu-Norberg H., Ouchida A.T., Norberg E. (2017). The role of mitochondria in metabolism and cell death. Biochem. Biophys. Res. Commun..

[19] De Carvalho C., Caramujo M.J. (2018). The Various Roles of Fatty Acids. Molecules.

[20] Talley J.T., Mohiuddin S.S. (2020). Biochemistry, Fatty Acid Oxidation.

[21] Pan T., Gao L., Wu G., Shen G., Xie S., Wen H., Yang J., Zhou Y., Tu Z., Qian W. (2015). Elevated expression of glutaminase confers glucose utilization via glutaminolysis in prostate cancer. Biochem. Biophys. Res. Commun..

[22] Fernie A.R., Carrari F., Sweetlove L.J. (2004). Respiratory metabolism: Glycolysis, the TCA cycle and mitochondrial electron transport. Curr. Opin. Plant Biol..

[23] Ashton T.M., McKenna W.G., Kunz-Schughart L.A., Higgins G.S. (2018). Oxidative Phosphorylation as an Emerging Target in Cancer Therapy. Clin. Cancer Res..

[24] Herzig S., Shaw R.J. (2018). AMPK: Guardian of metabolism and mitochondrial homeostasis. Nat. Rev. Mol. Cell Biol..

[25] Hardie D.G., Ross F.A., Hawley S.A. (2012). AMPK: A nutrient and energy sensor that maintains energy homeostasis. Nat. Rev. Mol. Cell Biol..

[26] Inoki K., Zhu T., Guan K.-L. (2003). TSC2 Mediates Cellular Energy Response to Control Cell Growth and Survival. Cell.

[27] Ahmadian M., Abbott M.J., Tang T., Hudak C.S., Kim Y., Bruss M., Hellerstein M.K., Lee H.-Y., Samuel V.T., Shulman G.I. (2011). Desnutrin/ATGL Is Regulated by AMPK and Is Required for a Brown Adipose Phenotype. Cell Metab..

[28] Wu N., Zheng B., Shaywitz A., Dagon Y., Tower C., Bellinger G., Shen C.-H., Wen J., Asara J., McGraw T.E. (2013). AMPK-Dependent Degradation of TXNIP upon Energy Stress Leads to Enhanced Glucose Uptake via GLUT1. Mol. Cell.

[29] Egan D.F., Shackelford D.B., Mihaylova M.M., Gelino S.R., Kohnz R.A., Mair W., Vasquez D.S., Joshi A., Gwinn D.M., Taylor R. (2010). Phosphorylation of ULK1 (hATG1) by AMP-Activated Protein Kinase Connects Energy Sensing to Mitophagy. Science.

[30] Hardie D.G., Schaffer B.E., Brunet A. (2016). AMPK: An Energy-Sensing Pathway with Multiple Inputs and Outputs. Trends Cell Biol..

[31] Kimmelman A.C., White E. (2017). Autophagy and Tumor Metabolism. Cell Metab..

[32] Kim J., Kundu M., Viollet B., Guan K.-L. (2011). AMPK and mTOR regulate autophagy through direct phosphorylation of Ulk. Nat. Cell Biol..

[33] González A., Hall M.N., Lin S.-C., Hardie D.G. (2020). AMPK and TOR: The Yin and Yang of Cellular Nutrient Sensing and Growth Control. Cell Metab..

[34] Lee C.H., Akin-Olugbade O., Kirschenbaum A. (2011). Overview of Prostate Anatomy, Histology, and Pathology. Endocrinol. Metab. Clin. N. Am..

[35] Bader D.A., McGuire S.E. (2020). Tumour metabolism and its unique properties in prostate adenocarcinoma. Nat. Rev. Urol..

[36] Gilany K., Minai-Tehrani A., Savadi-Shiraz E., Rezadoost H., Lakpour N. (2015). Exploring the Human Seminal Plasma Proteome: An Unexplored Gold Mine of Biomarker for Male Infertility and Male Reproduction Disorder. J. Reprod. Infertil..

[37] Costello L.C., Franklin R.B. (2008). Prostatic fluid electrolyte composition for the screening of prostate cancer: A potential solution to a major problem. Prostate Cancer Prostatic Dis..

[38] Costello L.C., Liu Y., Franklin R.B., Kennedy M.C. (1997). Zinc Inhibition of Mitochondrial Aconitase and Its Importance in Citrate Metabolism of Prostate Epithelial Cells. J. Biol. Chem..

[39] Costello L.C., Franklin R.B. (1998). Novel role of zinc in the regulation of prostate citrate metabolism and its implications in prostate cancer. Prostate.

[40] Kolenko V.M., Teper E., Kutikov A., Uzzo R.G. (2013). Zinc and zinc transporters in prostate carcinogenesis. Nat. Rev. Urol..

[41] Arver S. (1982). Zinc and zinc ligands in human seminal plasma. III. The principal low molecular weight zinc ligand in prostatic secretion and seminal plasma. Acta Physiol. Scand..

[42] Ford W.C.L., Harrison A. (1984). The role of citrate in determining the activity of calcium ions in human semen. Int. J. Androl..

[43] Twum-Ampofo J., Fu D.-X., Passaniti A., Hussain A., Siddiqui M.M. (2016). Metabolic targets for potential prostate cancer therapeutics. Curr. Opin. Oncol..

[44] Costello L.C., Franklin R.B., Zou J., Feng P., Bok R., Swanson M.G., Kurhanewicz J. (2011). Human prostate cancer ZIP1/zinc/citrate genetic/metabolic relationship in the TRAMP prostate cancer animal model. Cancer Biol. Ther..

[45] Costello L.C., Franklin R.B. (2006). The clinical relevance of the metabolism of prostate cancer; zinc and tumor suppression: Connecting the dots. Mol. Cancer.

[46] Zadra G., Photopoulos C., Loda M. (2013). The fat side of prostate cancer. Biochim. Biophys. Acta (BBA) Mol. Cell Biol. Lipids.

[47] Zou J., Milon B.C., Desouki M.M., Costello L.C., Franklin R.B. (2011). hZIP1 zinc transporter down-regulation in prostate cancer involves the overexpression of ras responsive element binding protein-1 (RREB-1). Prostate.

[48] Moon J.-S., Jin W.-J., Kwak J.-H., Kim H.-J., Yun M.-J., Kim J.-W., Park S.W., Kim K.-S. (2010). Androgen stimulates glycolysis for de novo lipid synthesis by increasing the activities of hexokinase 2 and 6-phosphofructo-2-kinase/fructose-2,6-bisphosphatase 2 in prostate cancer cells. Biochem. J..

[49] Bader D.A., Hartig S.M., Putluri V., Foley C., Hamilton M.P., Smith E.A., Saha P.K., Panigrahi A., Walker C., Zong L. (2019). Mitochondrial pyruvate import is a metabolic vulnerability in androgen receptor-driven prostate cancer. Nat. Metab..

[50] Costello L.C., Franklin R.B. (1993). Testosterone Regulates Pyruvate Dehydrogenase Activity of Prostate Mitochondria. Horm. Metab. Res..

[51] Wang Q., Hardie R., Hoy A.J., Van Geldermalsen M., Gao D., Fazli L., Sadowski M.C., Balaban S., Schreuder M., Nagarajah R. (2015). Targeting ASCT2 -mediated glutamine uptake blocks prostate cancer growth and tumour development. J. Pathol..

[52] White M.A., Lin C., Rajapakshe K., Dong J., Shi Y., Tsouko E., Mukhopadhyay R., Jasso D., Dawood W., Coarfa C. (2017). Glutamine Transporters Are Targets of Multiple Oncogenic Signaling Pathways in Prostate Cancer. Mol. Cancer Res..

[53] Heemers H., Maes B., Foufelle F., Heyns W., Verhoeven G., Swinnen J.V. (2001). Androgens stimulate lipogenic gene expression in prostate cancer cells by activation of the sterol regulatory element-binding protein cleavage activating protein/sterol regulatory element-binding protein pathway. Mol. Endocrinol..

[54] Swinnen J.V., Heemers H., Van De Sande T., De Schrijver E., Brusselmans K., Heyns W., Verhoeven G. (2004). Androgens, lipogenesis and prostate cancer. J. Steroid Biochem. Mol. Biol..

[55] Liby K.T., Yore M.M., Sporn M.B. (2007). Triterpenoids and rexinoids as multifunctional agents for the prevention and treatment of cancer. Nat. Rev. Cancer.

[56] Laszczyk M.N. (2009). Pentacyclic Triterpenes of the Lupane, Oleanane and Ursane Group as Tools in Cancer Therapy. Planta Medica.

[57] Ovesná Z., Vachálková A., Horváthová K., Tóthová D. (2004). *Pentacyclic triterpenoic* acids: New chemoprotective compounds. Minireview. Neoplasma.

[58] Gerhauser C. (2008). Cancer Chemopreventive Potential of Apples, Apple Juice, and Apple Components. Planta Medica.

[59] Silchenko A.S., Kalinovsky A.I., Avilov S.A., Andryjaschenko P.V., Dmitrenok P.S., Martyyas E.A., Kalinin V.I. (2013). Triterpene Glycosides from the Sea Cucumber *Eupentacta Fraudatrix*. Structure and Biological Action of Cucumariosides I1, I3, I4, Three New Minor Disulfated Pentaosides. Nat. Prod. Commun..

[60] Kolesnikova S.A., Lyakhova E.G., Kalinovsky A.I., Pushilin M.A., Afiyatullov S.S., Yurchenko E.A., Dyshlovoy S.A., Minh C.V., Stonik V.A. (2013). Isolation, Structures, and Biological Activities of Triterpenoids from a *Penares* sp. Marine Sponge. J. Nat. Prod..

[61] Liu X.-Y., Wang X.-L., Shen T., Ren D.-M., Lou H.-X. (2020). Two new triterpenoids from the fungus *Diplodia cupressi*. Nat. Prod. Res..

[62] Gao J.K.M., Zhang L., Bal L. (2020). Lokeshwar Spice up your food for cancer prevention: Cancer chemo-prevention by natural compounds from common dietary spices. Evolutionary Diversity as a Source for Anticancer Molecules.

[63] Li S., Kuo H.-C.D., Yin R., Wu R., Liu X., Wang L., Hudlikar R., Peter R.M., Kong A.-N. (2020). Epigenetics/epigenomics of triterpenoids in cancer prevention and in health. Biochem. Pharmacol..

[64] Phillips D.R., Rasbery J.M., Bartel B., Matsuda S.P. (2006). Biosynthetic diversity in plant triterpene cyclization. Curr. Opin. Plant Biol..

[65] Cunha L.C.S., Andrade e Silva M.L., Furtado N.A., Vinhólis A.H.C., Martins C.H.G., da Silva Filho A.A., Cunha W.R. (2007). Antibacterial activity of triterpene acids and semi-synthetic derivatives against oral pathogens. Z. Naturforsch C J. Biosci..

[66] Banno N., Akihisa T., Yasukawa K., Tokuda H., Tabata K., Nakamura Y., Nishimura R., Kimura Y., Suzuki T. (2006). Anti-inflammatory activities of the triterpene acids from the resin of *Boswellia carteri*. J. Ethnopharmacol..

[67] Parikh N.R., Mandal A., Bhatia D., Siveen K.S., Sethi G., Bishayee A. (2014). Oleanane triterpenoids in the prevention and therapy of breast cancer: Current evidence and future perspectives. Phytochem. Rev..

[68] Shanmugam M.K., Dai X., Kumar A.P., Tan B.K., Sethi G., Bishayee A. (2014). Oleanolic acid and its synthetic derivatives for the prevention and therapy of cancer: Preclinical and clinical evidence. Cancer Lett..

[69] Ghante M.H., Jamkhande P.G. (2019). Role of *Pentacyclic triterpenoids* in Chemoprevention and Anticancer Treatment: An Overview on Targets and Underling Mechanisms. J Pharmacopunct..

[70] Shanmugam M.K., Nguyen A.H., Kumar A.P., Tan B.K., Sethi G. (2012). Targeted inhibition of tumor proliferation, survival, and metastasis by *Pentacyclic triterpenoids*: Potential role in prevention and therapy of cancer. Cancer Lett..

[71] Guerra A.R., Duarte M.F., Duarte I.F. (2018). Targeting Tumor Metabolism with Plant-Derived Natural Products: Emerging Trends in Cancer Therapy. J. Agric. Food Chem..

[72] Pandita A., Kumar B., Manvati S., Vaishnavi S., Singh S.K., Bamezai R.N.K. (2014). Synergistic Combination of Gemcitabine and Dietary Molecule Induces Apoptosis in Pancreatic Cancer Cells and Down Regulates PKM2 Expression. PLoS ONE.

[73] Lewinska A., Adamczyk-Grochala J., Kwasniewicz E., Deregowska A., Wnuk M. (2017). Ursolic acid-mediated changes in glycolytic pathway promote cytotoxic autophagy and apoptosis in phenotypically different breast cancer cells. Apoptosis.

[74] Amara S., Zheng M., Tiriveedhi V. (2016). Oleanolic Acid Inhibits High Salt-Induced Exaggeration of Warburg-like Metabolism in Breast Cancer Cells. Cell Biophys..

[75] Lodi A., Saha A., Lu X., Wang B., Sentandreu E., Collins M., Kolonin M.G., DiGiovanni J., Tiziani S. (2017). Combinatorial treatment with natural compounds in prostate cancer inhibits prostate tumor growth and leads to key modulations of cancer cell metabolism. NPJ Precis. Oncol..

[76] Saha A., Blando J., Fernandez I., Kiguchi K., DiGiovanni J. (2016). Linneg Sca-1high CD49fhigh prostate cancer cells derived from the Hi-Myc mouse model are tumor-initiating cells with basal-epithelial characteristics and differentiation potential in vitro and In Vivo. Oncotarget.

[77] Liberti M.V., Locasale J.W. (2016). Correction to: ’The Warburg Effect: How Does it Benefit Cancer Cells?. Trends Biochem. Sci..

[78] Liu J., Wu N., Ma L., Liu M., Liu G., Zhang Y., Lin X. (2014). Oleanolic Acid Suppresses Aerobic Glycolysis in Cancer Cells by Switching Pyruvate Kinase Type M Isoforms. PLoS ONE.

[79] Dayton T.L., Jacks T., Heiden M.G.V. (2016). PKM 2, cancer metabolism, and the road ahead. EMBO Rep..

[80] Noguchi T., Inoue H., Tanaka T. (1986). The M1- and M2-type isozymes of rat pyruvate kinase are produced from the same gene by alternative RNA splicing. J. Biol. Chem..

[81] Parnell K.M., Foulks J.M., Nix R.N., Clifford A., Bullough J., Luo B., Senina A., Vollmer D., Liu J., McCarthy V. (2013). Pharmacologic Activation of PKM2 Slows Lung Tumor Xenograft Growth. Mol. Cancer Ther..

[82] Sun Q., Chen X., Ma J., Peng H., Wang F., Zha X., Wang Y., Jing Y., Yang H., Chen R. (2011). Mammalian target of rapamycin up-regulation of pyruvate kinase isoenzyme type M2 is critical for aerobic glycolysis and tumor growth. Proc. Natl. Acad. Sci. USA.

[83] David C.J., Chen M., Assanah M., Canoll P., Manley J.L. (2009). HnRNP proteins controlled by c-Myc deregulate pyruvate kinase mRNA splicing in cancer. Nat. Cell Biol..

[84] Liu J., Zheng L., Wu N., Ma L., Zhong J., Liu G., Lin X. (2014). Oleanolic Acid Induces Metabolic Adaptation in Cancer Cells by Activating the AMP-Activated Protein Kinase Pathway. J. Agric. Food Chem..

[85] Menendez J.A., Lupu R. (2007). Fatty acid synthase and the lipogenic phenotype in cancer pathogenesis. Nat. Rev. Cancer.

[86] Dinda B., Ghosh B., Arima S., Sato N., Harigaya Y. (2007). Chemical Constituents of *Evolvulus nummularius*. Indian J. Chem. B.

[87] Younis T., Khan M.R., Sajid M. (2016). Protective effects of *Fraxinus xanthoxyloides* (Wall.) leaves against CCl4 induced hepatic toxicity in rat. BMC Complement. Altern. Med..

[88] Younis T., Khan M.I., Khan M.R., Rasul A., Majid M., Adhami V.M., Mukhtar H. (2018). Nummularic acid, a triterpenoid, from the medicinal plant *Fraxinus xanthoxyloides*, induces energy crisis to suppress growth of prostate cancer cells. Mol. Carcinog..

[89] Akhtar N., Syed D.N., Khan M.I., Adhami V.M., Mirza B., Mukhtar H. (2015). The pentacyclic triterpenoid, plectranthoic acid, a novel activator of AMPK induces apoptotic death in prostate cancer cells. Oncotarget.

[90] Woods A., Johnstone S.R., Dickerson K., Leiper F.C., Fryer L.G., Neumann D., Schlattner U., Wallimann T., Carlson M., Carling D. (2003). LKB1 Is the Upstream Kinase in the AMP-Activated Protein Kinase Cascade. Curr. Biol..

[91] Zhang Y., Wang Y., Bao C., Xu Y., Shen H., Chen J., Yan J., Chen Y. (2012). Metformin interacts with AMPK through binding to gamma subunit. Mol. Cell. Biochem..

[92] Bello D., Webber M.M., Kleinman H.K., Wartinger D.D., Rhim J.S. (1997). Androgen responsive adult human prostatic epithelial cell lines immortalized by human papillomavirus 18. Carcinogenesis.

[93] Kaighn M.E., Narayan K.S., Ohnuki Y., Lechner J.F., Jones L.W. (1979). Establishment and characterization of a human prostatic carcinoma cell line (PC-3). Investig. Urol..

[94] Stone K.R., Mickey D.D., Wunderli H., Mickey G.H., Paulson D.F. (1978). Isolation of a human prostate carcinoma cell line (DU 145). Int. J. Cancer.

[95] Horoszewicz J.S., Leong S.S., Kawinski E., Karr J.P., Rosenthal H., Chu T.M., Mirand E.A., Murphy G.P. (1983). LNCaP model of human prostatic carcinoma. Cancer Res..

[96] Thalmann G.N., Anezinis P.E., Chang S.M., Zhau H.E., Kim E.E., Hopwood V.L., Pathak S., Von Eschenbach A.C., Chung L.W. (1994). Androgen-independent cancer progression and bone metastasis in the LNCaP model of human prostate cancer. Cancer Res..

[97] Wu T.T., Sikes R.A., Cui Q., Thalmann G.N., Kao C., Murphy C.F., Yang H., Zhau H.E., Balian G., Chung L.W.K. (1998). Establishing human prostate cancer cell xenografts in bone: Induction of osteoblastic reaction by prostate-specific antigen-producing tumors in athymic and SCID/bg mice using LNCaP and lineage-derived metastatic sublines. Int. J. Cancer.

[98] Sramkoski R.M., Pretlow T.G., Giaconia J.M., Pretlow T.P., Schwartz S., Sy M.S., Marengo S.R., Rhim J.S., Zhang D., Jacobberger J.W. (1999). A new human prostate carcinoma cell line, 22Rv1. In Vitro Cell. Dev. Biol. Anim..

[99] Lodi A., Saha A., Lu X., Wang B., Sentandreu E., Collins M., Kolonin M.G., DiGiovanni J., Tiziani S. (2017). Erratum: Combinatorial treatment with natural compounds in prostate cancer inhibits prostate tumor growth and leads to key modulations of cancer cell metabolism. NPJ Precis. Oncol..

[100] Kim J., DeBerardinis R.J. (2019). Mechanisms and Implications of Metabolic Heterogeneity in Cancer. Cell Metab..

[101] Wang Y., Ma S., Ruzzo W.L. (2020). Spatial modeling of prostate cancer metabolic gene expression reveals extensive heterogeneity and selective vulnerabilities. Sci. Rep..

[102] Carmona-Fontaine C., Deforet M., Akkari L., Thompson C.B., Joyce J.A., Xavier J.B. (2017). Metabolic origins of spatial organization in the tumor microenvironment. Proc. Natl. Acad. Sci. USA.

[103] Sharma H., Kumar P., Deshmukh R.R., Bishayee A., Kumar S. (2018). Pentacyclic triterpenes: New tools to fight metabolic syndrome. Phytomedicine.

[104] Pi J., Liu Z., Wang H., Gu X., Wang S., Zhang B., Luan H., Zhu Z. (2016). Ursolic Acid Nanocrystals for Dissolution Rate and Bioavailability Enhancement: Influence of Different Particle Size. Curr. Drug Deliv..

[105] Bishayee A., Ahmed S., Brankov N., Perloff M. (2011). Triterpenoids as potential agents for the chemoprevention and therapy of breast cancer. Front. Biosci..

